# Early Exercise-Based Rehabilitation for Patients with Acute Decompensated Heart Failure: A Systemic Review and Meta-Analysis

**DOI:** 10.31083/j.rcm2311356

**Published:** 2022-10-21

**Authors:** Lisong Liu, Jun Chen, Ning Zhao, Mingming Zhang, Lihui Zhou, Xiaoxia Ren, Ting Zhang, Pengcheng Zhao, Dayi Hu, Xingxue Pang, Zhongyi Jin

**Affiliations:** ^1^Cardiac Rehabilitation Center, Dongzhimen Hospital, Beijing University of Chinese Medicine, 100700 Beijing, China; ^2^Doctoral Department, Capital Medical University, 100069 Beijing, China; ^3^Department of Geriatrics, Chui Yang Liu Hospital Affiliated to Tsinghua University, 100021 Beijing, China; ^4^Department of Cardiology, Chui Yang Liu Hospital Affiliated to Tsinghua University, 100021 Beijing, China; ^5^Department of Cardiovascular Medicine, Dongzhimen Hospital, Beijing University of Chinese Medicine, 100700 Beijing, China; ^6^Department of Cardiology, Peking University People's Hospital, 100044 Beijing, China

**Keywords:** cardiac rehabilitation, heart failure, 6-minute walk distance, SPPB

## Abstract

**Background:**

Cardiac rehabilitation is an important part of the therapeutic regimen for chronic heart failure. Acute decompensated heart failure (ADHF) in hospitalized patients were usually excluded from cardiac rehabilitation programs. The initiation of cardiac rehabilitation with ADHF usually occurs after hospital discharge. This study included recent clinical trials in patients beginning early exercise-based rehabilitation during their hospitalization and compared the efficacy and safety of early cardiac rehabilitation to ADHF patients who didn’t receive cardiac rehabilitation.

**Methods:**

Clinical trials were searched from the EMBASE, PubMed, CENTRAL, and WAN FANG. We included randomized controlled trials (RCTs) in which early exercise-based rehabilitation started during the index hospitalization, from the establishment of the database to July 2022. RevMan 5.4 was used for the statistical analysis.

**Results:**

Six studies, with a total of 668 patients were included; 336 patients in the early rehabilitation group and 332 patients in the control group. Exercise capacity was significantly improved in the 6-minute walk distance [mean difference (MD): 32.97, 95% CI: 31.03 to 34.90, *p *< 0.00001], and the Short Physical Performance Battery (MD: 1.40, 95% CI: 1.35 to 1.44, *p *< 0.00001). The rate of all-cause rehospitalization was significantly decreased in the early rehabilitation group (OR: 0.67, 95% CI: 0.45 to 0.99, *p* = 0.04).

**Conclusions:**

Early exercise-based rehabilitation for eligible ADHF in-patients starting during, or early after, hospitalization could significantly improve exercise capacity. A transitional, individualized, progressive, exercise-based rehabilitation program during hospitalization combined with post-discharge clinic rehabilitation is an integrated rehabilitation strategy for acute decompensated heart failure.

## 1. Introduction

Acute decompensated heart failure (ADHF) usually occurs in patients with a history of heart failure (HF) and reduced left ventricular ejection fraction (LVEF). It is the main cause of unplanned hospitalizations and is associated with poor health-related quality of life, higher mortality, and an increased rate of rehospitalization [1]. Mortality or rehospitalization rates after one year following hospital discharge are increased from 25% to more than 45% [1]. The majority of patients with ADHF are elderly and frail, with severe impairments in strength, balance, mobility, and endurance [2]. The presence of these physical disabilities may explain the high rate of rehospitalization with ADHF, since most ADHF patients were rehospitalized not for recurrence of heart failure, but due to the comorbidities and complications resulting from these physical impairments [3].

Cardiac rehabilitation (CR) involves a comprehensive program in those patients with heart failure and a reduced ejection fraction (HFrEF; left ventricular ejection fraction [LVEF <40%]) [4]. Exercise-based rehabilitation is an important part of the treatment regimen and is a grade I A recommendation by the ESC clinical guidelines for chronic heart failure, which has been shown to improve exercise capacity, and quality of life, and reduce hospitalization for heart failure [1]. Current clinical management strategies for ADHF do not include patients with physical impairments, and these patients are usually excluded from in-patient cardiac rehabilitation programs. Recently, some studies had explored the efficacy and safety of transitional, individualized, and progressive early cardiac rehabilitation programs initiated during, or early after, hospitalization in ADHF patients [5, 6, 7, 8].

The present meta-analysis reviewed recent randomized controlled trials (RCTs) in which early exercise-based rehabilitation in patients with acute decompensated heart failure began during, or early after, hospitalization and compared the efficacy and safety of cardiac rehabilitation with patients who didn’t receive cardiac rehabilitation.

## 2. Method

### 2.1 Search Strategy 

This meta-analysis was conducted following the Preferred Reporting Items for Systematic Reviews and Meta-Analyses (PRISMA) extension for meta-analysis [9]. A comprehensive literature search was carried out on the PubMed, EMBASE, Cochrane Central Register of Controlled Trials (CENTRAL) in the Cochrane Library, and WAN FANG database using the keywords “acute heart failure”, “acute decompensated heart failure”, “cardiac rehabilitation”, “physical rehabilitation”, and “exercise training”. The literature search strategy is shown in **Supplementary Table 1**. We included articles for which a full text was available and no language limitations were imposed. We searched PROSPERO for similar systematic reviews in progress to avoid duplication, as well as ClinicalTrials.gov for ongoing studies. We contacted investigators or study sponsors of studies for which only the abstract was available and tried to obtain the full text; and excluded unpublished data. Two investigators independently screened all the retrieved titles, abstracts, and selected articles for further screening. Disagreements were resolved by discussion.

### 2.2 Literature Inclusion and Exclusion Criteria 

Randomized controlled trials (RCTs) that focused on the efficacy and safety of patients with ADHF who underwent exercise-based early cardiac rehabilitation starting from hospitalization, written in English or Chinese up to July 2022 were included in this meta-analysis.

The criteria for considering RCTs were: (1) adults hospitalized due to a diagnosis of ADHF including preserved EF or reduced EF, (2) rehabilitation programs targeting physical activity or exercise training (compared with usual care and/or education), initiated in the hospital, and (3) report of numerical data for physical activity outcomes at completion for both groups. Physical activity outcome was defined as any bodily movement produced by skeletal muscles that require energy expenditure.

The exclusion criteria were: (1) retrospective clinical trials, (2) controls undertaken simultaneously with other exercise-based rehabilitation programs, (3) patients with stimulation of muscles of both legs, (4) insufficient data, and (5) patients receiving invasive or non-invasive assist devices for the duration of cardiac rehabilitation.

### 2.3 Data Extraction and Outcome Measures

All relevant studies were imported into a reference manager software program (EndNote x8.1, Thomson Reuters, Stanford, Connecticut, USA). We designed and utilized a data collection form to extract data on study characteristics and outcomes. After duplicate removal, two investigators (JC, and ZYJ) reviewed the full text and extracted the following data from each included article: (a) General information: study name (author, year), study design, duration of cardiac rehabilitation; (b) Participant information: number of participants, diagnosis, inclusion criteria; (c) Interventions: characteristics of exercise based-rehabilitation; (d) Outcomes: exercise capacity, quality of life and adverse events during the cardiac rehabilitation; Disagreements (if any) were resolved by discussion.

### 2.4 Quality Assessment

The quality assessment was carried out by the Cochrane Handbook for Systematic Review of Intervention-version 5.1.0 recommended risk assessment tool for bias in RCTs [10]. The assessment content included the following 7 items: (I) which random method to use; (II) whether to perform allocation concealment; (III) the implementation of blinding between patients and investigators; (IV) the effect of blinding; (V) whether the results were complete; (VI) whether the survey results were credible; and (VII) other biases.

### 2.5 Data Analysis 

A Forest-plot map clearly showed the results of each study. The absence of an overlap between the confidence intervals (CIs) of the results of each study indicated no statistical homogeneity between the studies.

### 2.6 Sensitivity Analysis

The sensitivity analysis investigated whether a single study affected the overall results of the combined data set, which would have an impact on the outcomes in the following situations: (1) when a study was deleted, the result will be significantly different. If there was little difference in the overall results when a study was deleted, it indicated the sensitivity of the combined results and the results obtained were unstable. (2) The results showed sensitivity and stability, and the conclusion was correct.

### 2.7 Statistical Analysis

The meta-analysis was performed using the RevMan 5.4 software. For continuous variables, the mean difference (MD) was used when the outcomes of the included studies were measured using the same methodology; otherwise, the standardized mean difference (SMD) was used when studies assessed the same outcome with different methodologies [10]. For Dichotomous variables, the odds ratio (OR) was used as the effect size, and the 95% CI expressed the results. The included studies were first tested for heterogeneity, with α = 0.1 as the test level. If there was no heterogeneity between the studies (*p *> 0.1, $I^{2}$ < 50%), the fixed-effects model (FEM) was used for the meta-analysis; if there was heterogeneity between studies ($I^{2}$ < 50%), the random-effects model (REM) was used [11]. *p *< 0.05 indicated that the difference was statistically significant. When the meta-analysis comparison of one endpoint index included more than 10 articles, a funnel chart was used to analyze the publication bias [12].

## 3. Results

### 3.1 Search Results

**Fig. 1.**
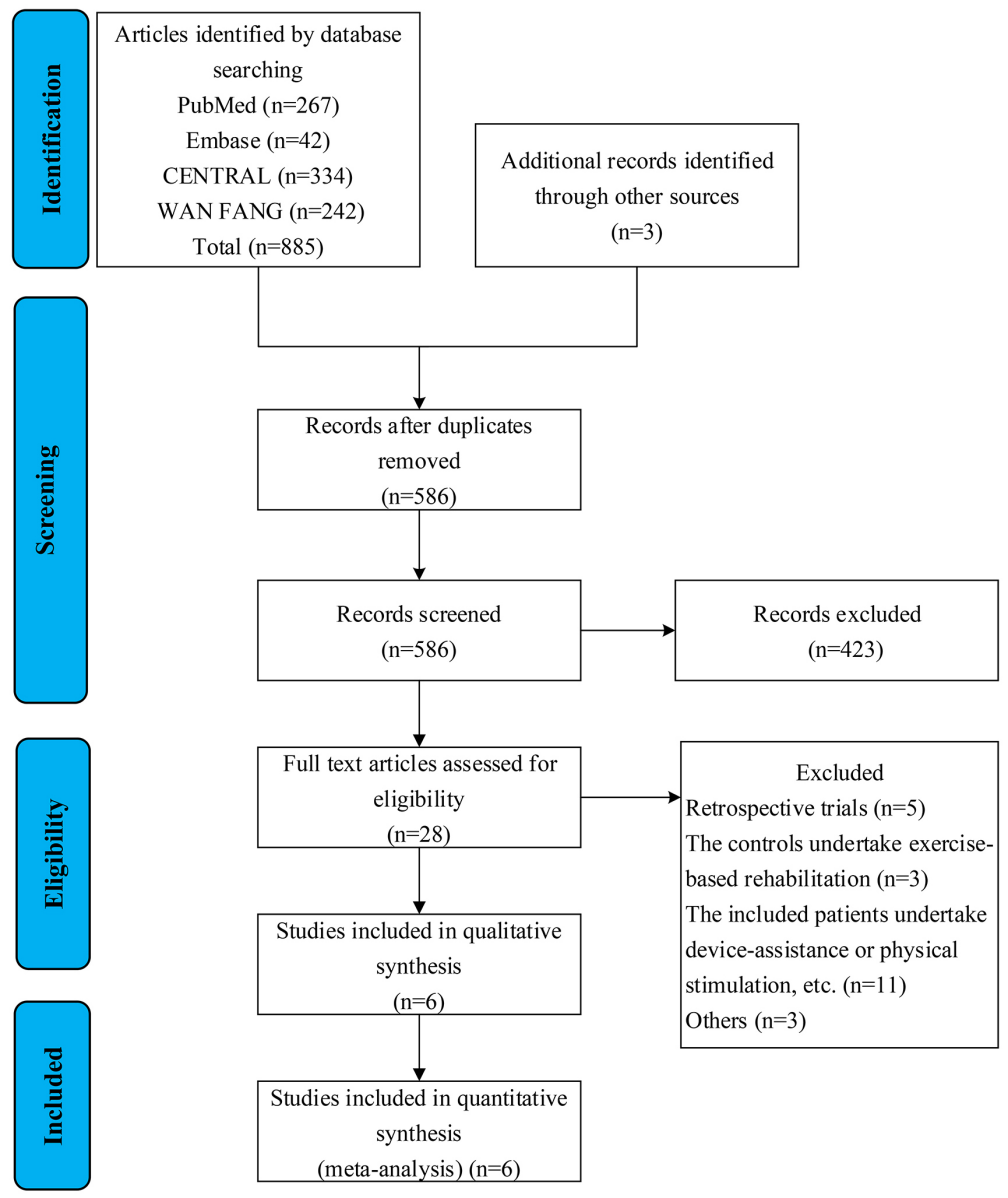
**Selection flow chart of literature screening**.

Search results and reasons for exclusion are listed in the Preferred Reporting Items for Systematic Reviews and meta-analyses diagram (Fig. 1). A total of six trials evaluating early exercise-based rehabilitation initiated during the hospitalization [5, 6, 7, 8, 13, 14] and at 6 months (n = 3) follow-up were included in the meta-analysis. We also included one study in which the patients in the exercise-training group and the controls were involved in the pooled analysis, but the data from the group with exercise-training receiving non-invasive devices was excluded due to our previously cited exclusion criteria (Table 1, Ref. [5, 6, 7, 8, 13, 14]).

**Table 1.** **Characteristics of the included clinical trials**.

Author (Year)	Country	Study design	Sample size, n (CR/Control)	Female, % (CR/Control)	Age, years (CR/Control)	Inclusion criteria	Timing of initiation and duration	Duration	Endpoint measurement
Babu *et al*., 2011 [13]	India	RCTs	30 (15/15)	20%/33%	56.87 ± 10.45/58.73 ± 10.81	ADHF; NYHA class II–IV	Hospital stay	8 weeks	6MWD, SF36
Oliveira *et al*., 2017 [14]	Brazil	RCTs	18 (9/9)	22%/11%	58 ± 7/57 ± 5	ADHF; LVEF <30%; NYHA class IV.	Hospital stay	Hospital stay	FEV1, % predicted; NYHA function; NT-proBNP; 6MWD
Reeves *et al*., 2017 [8]	USA	RCTs	27 (15/12)	47%/33%	72.7 ± 10.8/71.8 ± 9.1	ADHF regardless of ejection fraction; >60 yr,	Hospital stay	3 months	SPPB, rate of rehospitalization; 6MWD; Quality of life
Delgado *et al*., 2020 [6]	Portuga	RCTs	100 (50/50)	30%/40%	69.3 ± 9.5/70.3 ± 10.5	ADHF; >18 yr	Hospital stay	Hospital stay, 10 days	6MWD, Barthel Index (BI)
Kitzman *et al*., 2021 [5]	USA	RCTs	349 (175/174)	49%/56%	73.1 ± 8.5 /72.2 ± 7.7	ADHF regardless of ejection fraction; walk at least 4 m at enrollment; ≥60 yr	Hospital stay	3 months	SPPB; rate of rehospitalization; 6MWD; Quality of life; frailty status
Zhong hui *et al*., 2021 [7]	China	RCTs	144 (72/72)	48.6%/35.8%	75.3 ± 9.52 /75.5 ± 8.89	ADHF; ≥60 yr; at least 24 h of admission	Hospital stay	3 months	SPPB

Values shown are n (%), mean ± SD, or median (interquartile range). ADHF, acute decompensated heart failure; NYHA class, New York Heart Association class; CR, cardiac rehabilitation; LVEF, left ventricular ejection fraction; 6MWD, six-minute walk distance; SPPB, Short Physical Performance Battery; ET, exercise training; BI, Barthel index; KCCQ, Kansas City Cardiomyopathy Questionnaire.

### 3.2 Description of Included Clinical Trials

The trials were conducted between 2011 and 2021. The trials included a total of 668 patients. A large proportion (52.2%) of participants were drawn from the REHAB-HF trial (n = 349) (Tables 1,2,3, Ref. [5, 6, 7, 8, 13, 14]).

**Table 2.** **In-patient rehabilitation protocol of the included clinical trials**.

Author (Year)	Type	Target frequency	Target intensity	Target duration	Individualized evaluation	Supervised rehabilitation
Babu *et al*., 2011 [13]	Four-step Aerobic exercise	Once a day during hospitalization if tolerated	Modified Borg’s RPE between 3–4/10 individual adjustment	10 minutes/per session, if tolerated	Yes	Yes
Oliveira *et al*., 2017 [14]	Aerobic exercise (unloaded in-bed cycle ergometer combines non-invasive ventilation)	Once a day during hospitalization if tolerated	Initiated from a low level, individualized adjustment	20 minutes/per session, if tolerated	Yes	Yes
Reeves *et al*., 2017 [8]	Strength, endurance, mobility, and balance based on patient functional level in each category	Once a day during hospitalization if tolerated	RPE (6–20 scale) and initiated from low (≤12); after 2 weeks increased to 13 (“somewhat hard”; range 11–15) for endurance training and 15–16 for strength training	30-minutes/per session, if tolerated	Yes	Yes
Delgado *et al*., 2020 [6]	Aerobic exercise was divided into five stages of progressive levels of intensity	At least 5 days per week, twice a day (average of 10 sessions per week)	SPE ≤8, initiated from a low level, individualized adjustment	5–20 minutes/ per session, if tolerated	Yes	Yes
Kitzman *et al*., 2021 [5]	Strength, endurance, mobility, and balance based on patient functional level in each category.	once per day	RPE ≤12, initiated from a low level, individualized adjustment	approximately 45 min/per session, if tolerated	Yes	Yes
Zhong hui *et al*., 2021 [7]	Strength, endurance, mobility, and balance based on patient functional level in each category.	3 times/per week	RPE ≤12, initiated from a low level	30min/per session, if tolerated	Yes	Yes

SPE, modified Borg scale of subjective perceived exertion; RPE, rate of perceived exertion.

**Table 3.** **Out-patient rehabilitation protocol of the included studies**.

Author (Year)	Type	Target frequency	Target intensity	Target duration	Individualized evaluation	Supervised rehabilitation
Babu *et al*., 2011 [13]	Walking program	Once a day	RPE ≤7	5–40 minutes/per session, if tolerated, 8 weeks	Yes	Yes
Oliveira *et al*., 2017 [14]	N/A	N/A	N/A	N/A	N/A	N/A
Reeves *et al*., 2017 [8]	Strength, endurance, mobility, and balance based on patient functional level in each category.	3 times/per week	Endurance training, RPE 13 (11–15); strength training, RPE 15–16	60 minutes/per session, if tolerated, 12 weeks	Yes	Yes
Delgado *et al*., 2020 [6]	N/A	N/A	N/A	N/A	N/A	N/A
Kitzman *et al*., 2021 [5]	Strength, endurance, mobility, and balance based on patient functional level in each category.	3 times/per week	Endurance training, RPE 13 (11–15); strength training, RPE 15–16	60 minutes/per session, if tolerated, 12 weeks	Yes	Yes
Zhong hui *et al*., 2021 [7]	Strength, endurance, mobility, and balance based on patient functional level in each category.	3 times/per week	RPE ≤12, gradually increased up to RPE 13, but less than 15	60 minutes/per session, if tolerated, 12 weeks	Yes	Yes

SPE, modified Borg scale of subjective perceived exertion; RPE, rate of perceived exertion.

### 3.3 Early Exercise-Based Rehabilitation

The composition of rehabilitation protocols in the included studies differed in target duration, intensity, mode, frequency, and function domain (Tables 2,3).

### 3.4 6-Minute Walk Distance

**Fig. 2.**
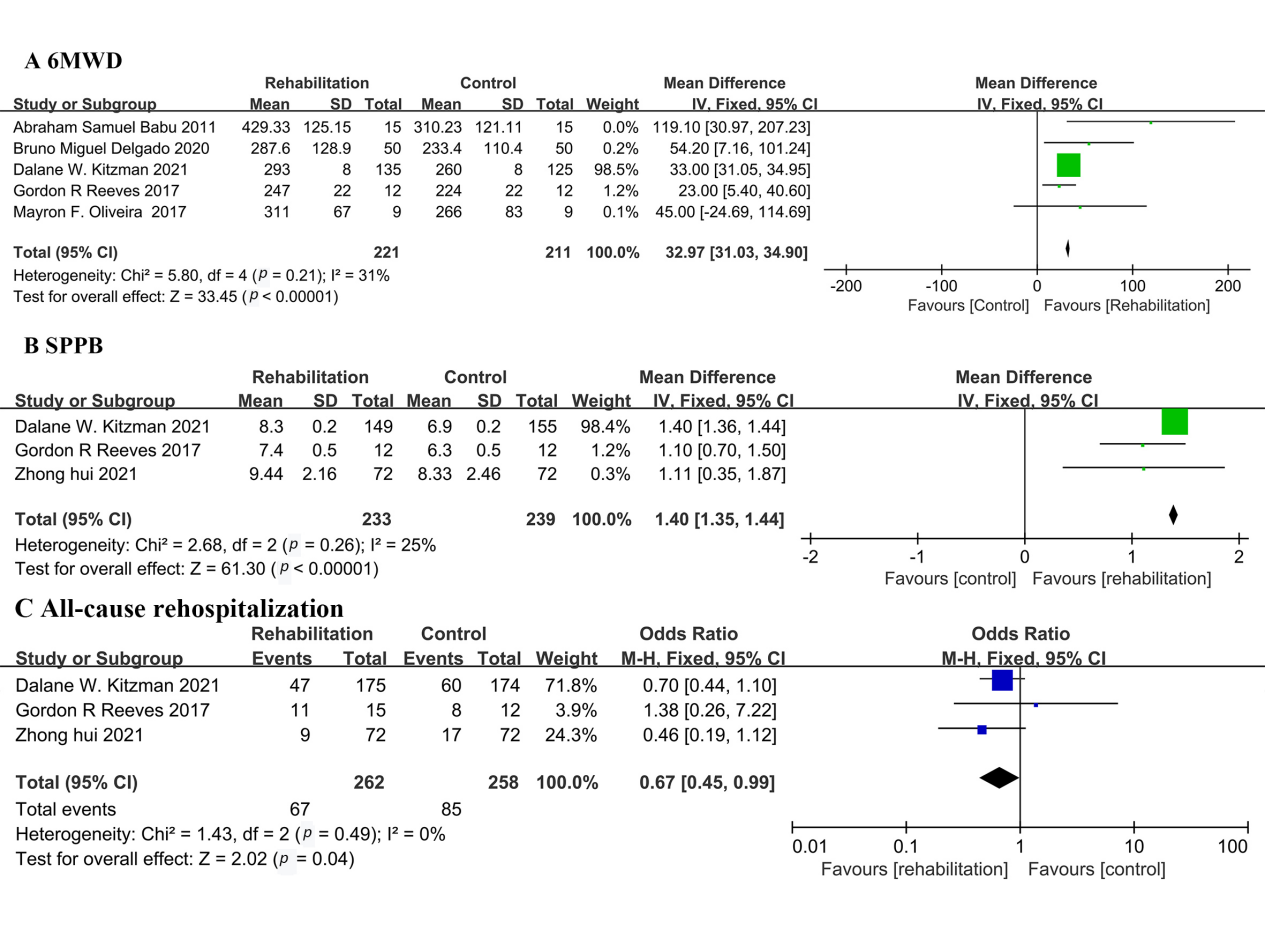
**Forest plot illustrating a comparison of the efficacy between early exercise-based rehabilitation and the control**. (A) 6-minute walk distance. (B) Short Physical Performance Battery (SPPB). (C) Quality of Life.

Five studies reported the six-minute walk distance in patients at the end of early exercise-based rehabilitation [5, 6, 8, 13, 14]. The duration of rehabilitation ranged from 10 days up to 3 months in these studies. The heterogeneity test showed that $I^{2}$ = 31%, and *p* = 0.21, which indicated that there was no obvious heterogeneity. The FEM was then used for analysis which showed that MD was 32.97 m, 95% CI: 31.03 to 34.90, Z = 33.45, and *p *< 0.00001 (Fig. 2).

### 3.5 Short Physical Performance Battery 

Three studies reported the Short Physical Performance Battery (SPPB) of patients undergoing 3-months of cardiac rehabilitation [5, 7, 8]. The heterogeneity test showed that $I^{2}$ = 25% and *p* = 0.26, which indicated that there was no obvious heterogeneity. The FEM showed that MD was 1.40, 95% CI: 1.35 to 1.44, Z = 61.3, and *p *< 0.00001 (Fig. 2).

### 3.6 All-Cause Rehospitalization

Three articles reported all-cause rehospitalization [5, 7, 8]. The heterogeneity test showed that $I^{2}$ = 0% and *p* = 0.49, which indicated that there was obvious heterogeneity. The REM was used for analysis and showed that OR was 0.67, 95% CI: 0.45 to 0.99, Z = 2.02, and *p* = 0.04 (Fig. 2).

### 3.7 Falls or Injuries 

Four articles reported falls or injuries during early exercise-based rehabilitation [5, 6, 8, 14]. Falls and injury was present in the patients of only one study. The other three studies reported no events. Thus, there was no obvious heterogeneity. The REM was then used for analysis. The meta-analysis results showed that OR: 0.68, 95% CI: 0.43 to 1.08, Z = 1.65, and *p* = 0.10 (**Supplementary Fig. 2**).

### 3.8 Chest Pain

Four studies reported chest pain during early exercise-based rehabilitation [5, 6, 8, 14]. Chest pain was present in the patients of two studies. The other two studies reported no events. The heterogeneity test showed that $I^{2}$ = 0% and *p* = 0.83, which indicated that there was no obvious heterogeneity. The FEM was then used for the analysis. The meta-analysis results showed that OR: 1.86, 95% CI: 0.58 to 5.97, Z = 1.05, and *p* = 0.29 (**Supplementary Fig. 2**).

### 3.9 All-Cause Death

Three articles reported the rate of all-cause death at 6 months follow-up [5, 7, 8]. The all-cause death was present in the patients of one study and the incidence in the other two articles was zero, where the rate of death was not significantly different in the rehabilitation arm compared to placebo (OR: 1.35, 95% CI: 0.68 to 2.68; Z = 0.85, and *p* = 0.40) (**Supplementary Fig. 2**).

### 3.10 Quality of Life, Components of SPPB (See Supplementary Materials)

In terms of quality of life and components of SPPB, the early rehabilitation group also showed significant improvement compared with the controls. see **Supplementary Materials**.

### 3.11 Assessment of Risk Bias in the Included Studies

**Fig. 3.**
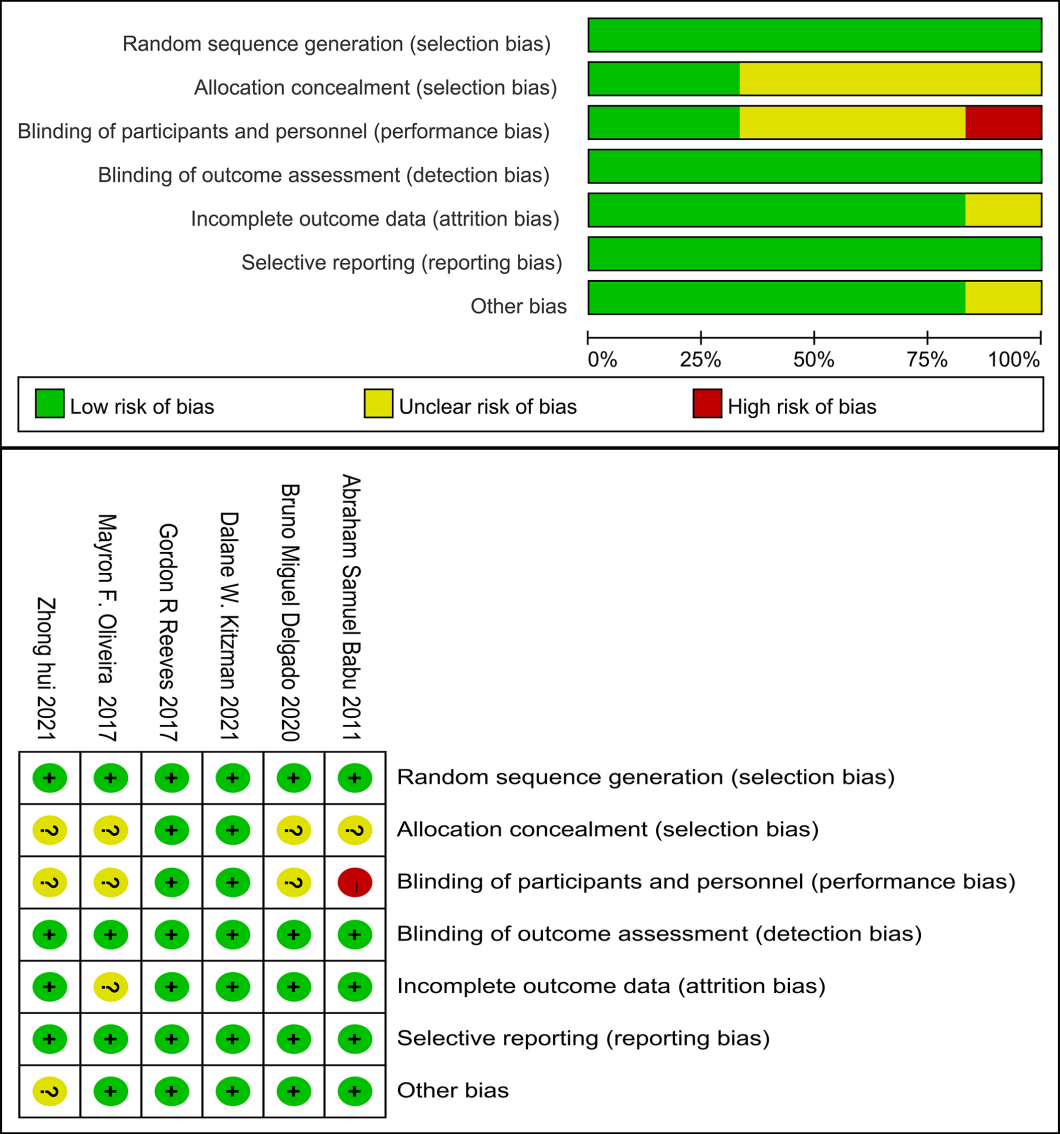
**Assessment of probable bias by the included trials**.

The Cochrane Handbook for Systematic Review of Intervention-version 5.1.0 was used to assess the risk of bias in the 6 articles included in the present study. The risk of bias was expressed using RevMan 5.4 software (Fig. 3).

## 4. Discussion

Six studies were included in this meta-analysis. All 6 studies used random grouping to divide the patients into early exercise-based rehabilitation groups, and controls or usual care groups which did not receive any exercise training by clinicians. Among these studies, there were some differences in rehabilitation protocols, duration of rehabilitation, and assessment of the quality of life. However, to our knowledge, the present study is the first meta-analysis evaluating early exercise-based rehabilitation for hospitalized patients with ADHF, including those beginning rehabilitation shortly after hospitalization. Our findings demonstrated that early exercise-based rehabilitation was safe and significantly improved physical function as assessed by 6MWD and the short physical performance battery (SPPB) in the patients with ADHF.

Physical disabilities, frailty, and depression are often clinically unrecognized in older patients hospitalized for acute heart failure and are generally not addressed in clinical care pathways, and may contribute to delayed, incomplete recovery and higher rates of rehospitalization, death, and long-term loss of independence after hospital discharge. Patients with acute heart failure, including those who were able to walk independently before hospitalization, spend most of their time in bed during hospitalization [15]. Long-term bed rest worsens physical function and increases the risk of cognitive decline and psychological disorders [16]. The cause of inactivity during hospitalization is multi-factorial. Cardiologists are usually concerned about the safety of moving patients out of bed too early in order to prevent falls, as well as the traditional concept that patients should stay in bed when they have a serious illness [17]. Studies have shown that the mean baseline 6-minute walk distance is less in acute decompensated heart failure compared to chronic heart failure. In addition, severe leg weakness does not allow standing from a seated position without the use of the patient’s arms [18].

Cardiac rehabilitation is an important part of the comprehensive therapeutic regimen for chronic heart failure (HFrEF) and has been shown to significantly improve physical function, exercise capacity, quality of life, and reduces hospitalizations [19, 20, 21]. Currently, exercised-based rehabilitation for heart failure is usually initiated in the period after hospital discharge when physical dysfunction is improved. Previous studies of rehabilitation commonly exclude those patients who had been hospitalized within six weeks from discharge [18, 22]. The evidence for beginning rehabilitation during this period was unclear. Kitzman *et al*. [5] demonstrated that patients with ADHF who undertook early exercise-based training significantly improved the short physical performance battery (SPPB) and 6MWD, and that the procedure was relatively safe. SPPB is a comprehensive measure in elderly patients to evaluate muscle function and strength [23]. Previous studies had shown that poor SPPB is closely associated with frailty in elderly hospitalized patients with cardiovascular disease, and is an independent risk factor for frailty, which can accurately predict patient disability, rehospitalization, home treatment, and death [24, 25]. Our pooled analysis of included studies also demonstrates that patients who undertake early exercise-based rehabilitation have significant improvement in physical function compared with those patients who undertake usual care without any other rehabilitation training. Quality of life assessments including the Kansas City Cardiomyopathy Questionnaire (KCCQ), the Short Form-36 Health Survey (SF-36) [26], and the Barthel Index (BI) [27] also improved in our meta-analysis and was consistence with improvement in physical function.

Rehabilitation programs should be individualized for every patient based on basic performance level. Most ADHF patients will begin at a low level, since they present frailty and a lower level of physical function. Kitzman *et al*. [5] reported that the exercise protocol should be analyzed by multiple domains including endurance, strength, balance, and flexibility. The initiation of rehabilitation for most patients was usually in the low level after individualized assessment of these domains. For example, intensity was determined by the patient’s rate of perceived exertion (RPE) and the target for inpatients was RPE ≤12 [5]. Balance and flexibility are usually in the lower level at baseline and the deficits in balance, flexibility, and mobility are more likely to lead to falls and injuries following the initiation of strength and endurance training, however, it is less common in chronic heart failure. Thus, training for balance and flexibility should be strengthened [5]. In Hui Zhong’s rehabilitation protocol, the patients with cardiac function IV in the four-step protocol received exercise training in bed, including breathing exercises and ankle foot movements, rather than out of bed [13].

Although the duration of these early rehabilitation protocols differed, four out of six included studies reported early rehabilitation during hospitalization combined with clinic rehabilitation. Cardiac rehabilitation during, or early after, hospitalization combined with rehabilitation for a period after hospital discharge mirrors the pathophysiological process of heart failure from the time of acute decompensation, through the transition to recovery, and finally to the return to a chronic, stable stage. Thus, this rehabilitation process from hospitalization to the clinic for acute decompensated heart failure should be more utilized in clinical practice.

However, it should be noted that not all in-patients with ADHF are suitable for early exercise-based rehabilitation, especially those in-patients with severe lower limb weakness and balance problems [28]. If a patient falls and is injured, the patient’s willingness to participate in cardiac rehabilitation is decreased. The rate of falls or injuries, and chest pain during exercise training were not significantly different in the included studies in this meta-analysis, which may have been due to the improved exercise capacity of these patients at baseline and the tailored rehabilitation protocol that was instituted. The patients who undertake the REHAB-HF protocol can walk at least 4 m at enrollment and an individualized rehabilitation protocol is based on an assessment of physical function in the four-domains at baseline. These inclusion criteria are the foundation for a successful exercise training program in ADHF patients. Furthermore, the exercise rehabilitation programs should be supervised by a rehabilitation clinician in a monitored environment to ensure that the process of supervised rehabilitation is relatively safe.

The clinical evidence that early transitional rehabilitation during hospitalization for patients with ADHF is safe and effective remains limited and more data is need to determine its long-term benefits. Early transitional rehabilitation for ADHF still faces great challenges, and requires the cooperation of patients, nurses, and physicians. However, our findings lend further support for the concept that rehabilitation should begin in the hospital. Our meta-analysis suggests that early exercise-based rehabilitation is an important treatment for ADHF. Proper selection of patients, supervised rehabilitation protocols, and individualized patient assessment are the key elements for a successful program. A transitional, individualized, progressive exercise-based rehabilitation program initiated during hospitalization combined with home-based or clinic rehabilitation is an integrated rehabilitation strategy for acute decompensated heart failure.

## 5. Limitation

The limitations of this study should be acknowledged. First, the number of included studies and sample size of the analysis are small, which reflects the limited number of studies on early rehabilitation in ADHF. However, this increases the risk of selection bias. Second, studies with device-assisted rehabilitation methods were excluded, such as electrical muscle stimulation and non-invasive ventilation. Third, the duration of rehabilitation included in this study varied from at least ten days to three months, which also increases the risk of bias. Fourth, the rehabilitation protocols varied amongst the studies. Finally, although blinding was used in the included studies, and the staff members who assessed the primary outcome were unaware of the trial group assignments, it was not possible for patients to be unaware of the group to which they had been randomly assigned; which also increased the risk of bias.

## 6. Conclusions

This meta-analysis demonstrated that early exercise-based rehabilitation for eligible in-patients with ADHF starting during, or early after, hospitalization, is relatively safe and could improve physical function. For patients with acute decompensated heart failure, a transitional, individualized, progressive exercise-based rehabilitation program during hospitalization combined with home-based or clinic rehabilitation is an integrated rehabilitation strategy.

## Abbreviations

ADHF, Acute Decompensated Heart Failure; CR, Cardiac Rehabilitation; SPPB, Short Physical Performance Battery; EF, Ejection Fraction; LVEF, Left Ventricular Ejection Fraction; 6MWD, 6-Minute Walk Distance; RPE, Rate of perceived exertion.

## Supplementary Material

Supplementary material associated with this article can be found, in the online version, at https://doi.org/10.31083/j.rcm2311356.
