# Supplementary material for: Early Exercise-Based Rehabilitation for Patients with Acute Decompensated Heart Failure: A Systemic Review and Meta-Analysis
Source: Rev Cardiovasc Med. 2022 Oct 21;23(11):356. doi: 10.31083/j.rcm2311356 (PMC11269056; doi:10.31083/j.rcm2311356)
Supplement: Supplementary file 1 [file 2153-8174-23-11-356-s1.doc]

Supplementary Materials

# 1. Supplementary results

## Components of SPPB

Three articles reported the balance score at the end of 3-months of early exercise-based rehabilitation. The heterogeneity test showed that I^2^ = 0%, and P = 0.43, which indicated that there was no obvious heterogeneity. The FEM was then used for analysis. The meta-analysis results showed that MD: 0.3, 95% CI: 0.28 to 0.32, Z = 26.61, and P<0.00001. (supplementary figure 1)

Three articles reported the chair rise score at the end of 3-months of early exercise-based rehabilitation. The heterogeneity test showed that I^2^ = 0%, and P = 0.77, which indicated that there was no obvious heterogeneity. The FEM was then used for analysis. The meta-analysis results showed that MD: 0.60, 95% CI: 0.58 to 0.62, Z = 52.68, and P<0.00001. (supplementary figure 1)

Two articles reported the 4-meter walk score at the end of 3-months of early exercise-based rehabilitation. The heterogeneity test showed that I^2^ = 0%, and P = 0.67, which indicated that there was no obvious heterogeneity. The FEM was then used for analysis. The meta-analysis results showed that MD: 0.50, 95% CI: 0.48 to 0.52, Z = 43.69, and P<0.00001. (supplementary figure 1)

## Quality of life

Four studies reported the quality of life of patients during early exercise-based rehabilitation. Two studies used the Kansas City Cardiomyopathy Questionnaire (KCCQ), one study used the Short Form-36 Health Survey (SF-36) and the rest used the Barthel Index (BI). The heterogeneity test showed that I^2^ = 97% and P<0.0001, which indicated that there was obvious heterogeneity. The REM was then used for analysis. The meta-analysis results showed that SMD: 2.44, 95% CI: 0.7 to 4.18, Z = 2.75, and P=0.006. (supplementary figure 3)

# 2. Supplementary figure legend


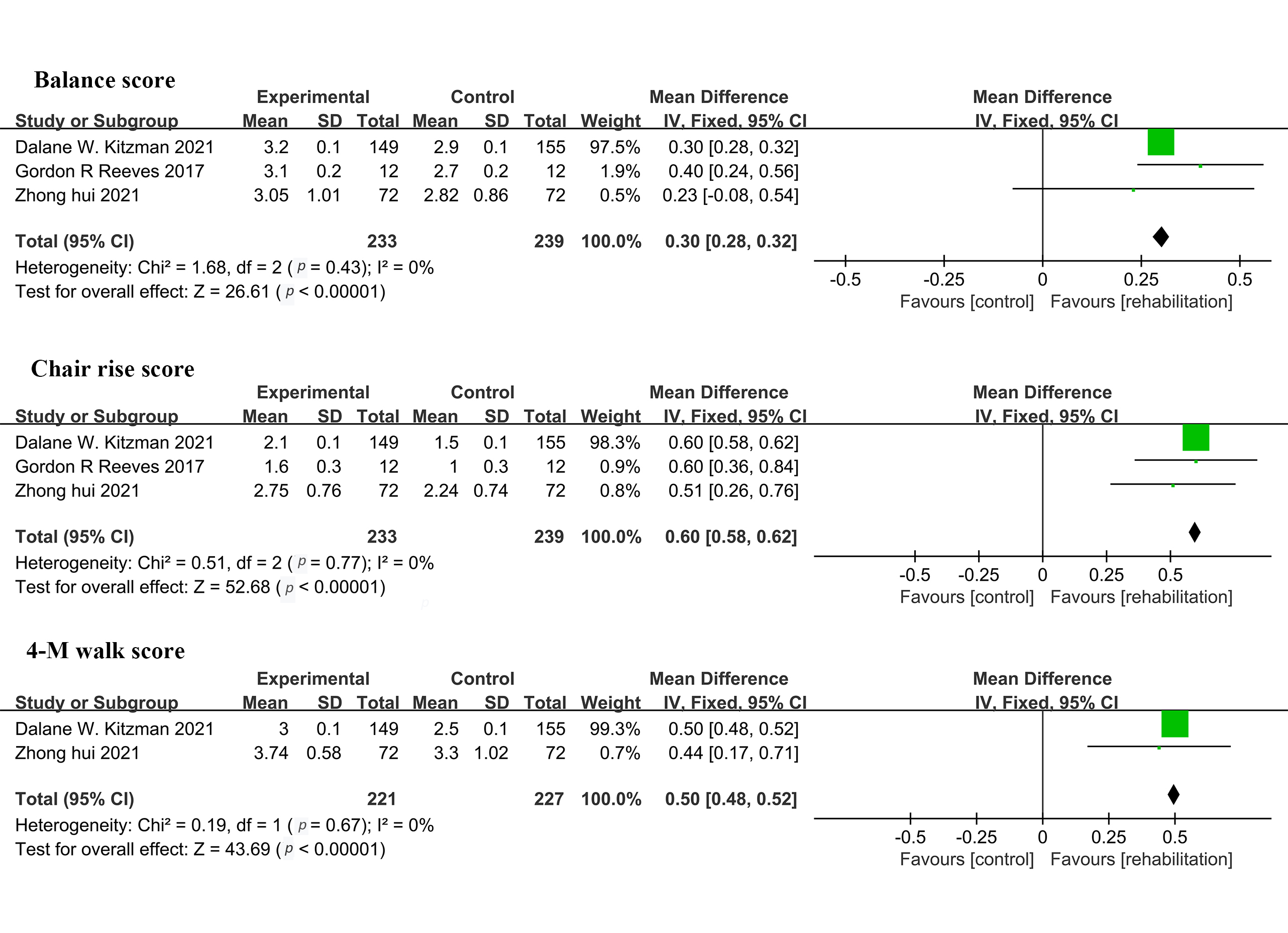


**Supplementary Fig. 1. Forest plot illustrating a comparison of the three components (balance score, chair rise score, and 4-meter walk score) of the *Short Physical Performance Battery* between early exercise-based rehabilitation and the control.**


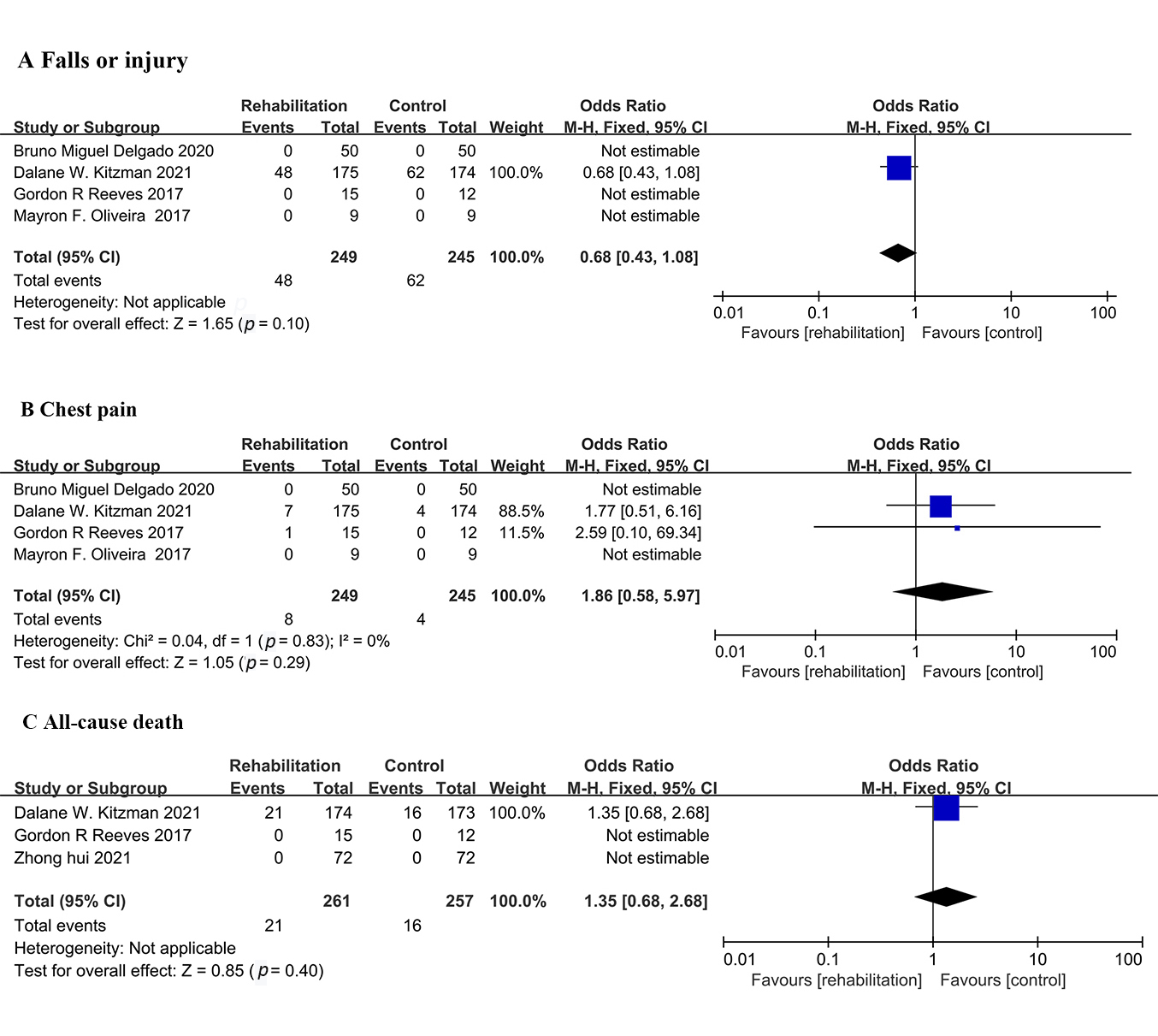


**Supplementary Fig. 2. Forest plot illustrating a comparison of safety between early exercise-based rehabilitation and control.** A. fall or injury during rehabilitation; B. chest pain during rehabilitation; C. all-cause death.


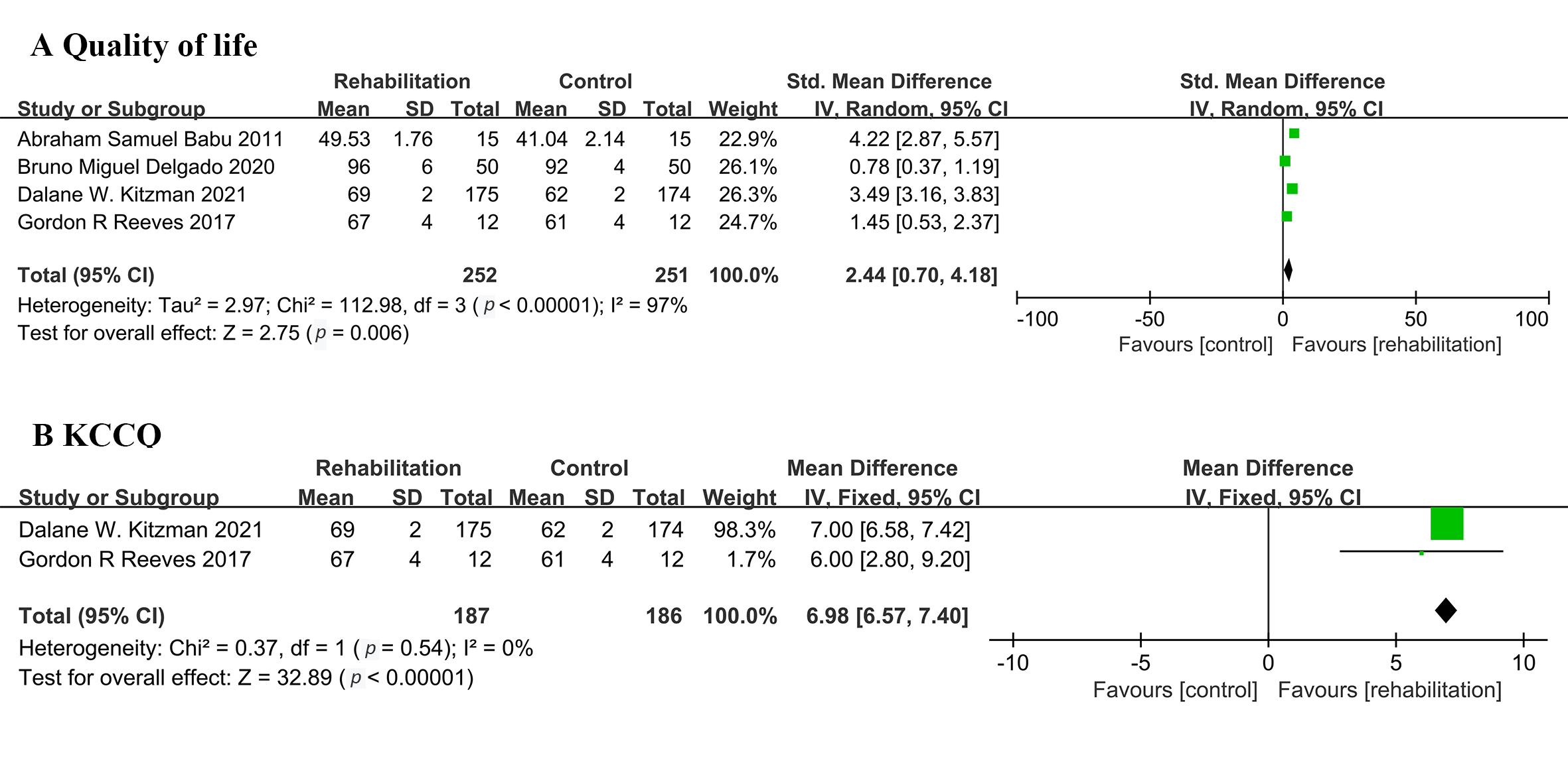


**Supplementary Fig. 3. Forest plot illustrating a comparison of quality of life.** A. quality of life B. KCCQ.

Supplementary Table 1. Research strategy.

| Pubmed | Cardiac rehabilitation | #1 " (("physical examination"[MeSH Terms] OR ("physical"[All Fields] AND "examination"[All Fields]) OR "physical examination"[All Fields] OR "physical"[All Fields] OR "physically"[All Fields] OR "physicals"[All Fields]) AND ("rehabilitant"[All Fields] OR "rehabilitants"[All Fields] OR "rehabilitate"[All Fields] OR "rehabilitated"[All Fields] OR "rehabilitates"[All Fields] OR "rehabilitating"[All Fields] OR "rehabilitation"[MeSH Terms] OR "rehabilitation"[All Fields] OR "rehabilitations"[All Fields] OR "rehabilitative"[All Fields] OR "rehabilitation"[MeSH Subheading] OR "rehabilitation s"[All Fields] OR "rehabilitational"[All Fields] OR "rehabilitator"[All Fields] OR "rehabilitators"[All Fields])) OR ("exercise"[MeSH Terms] OR "exercise"[All Fields] OR ("exercise"[All Fields] AND "training"[All Fields]) OR "exercise training"[All Fields]) OR ("cardiac rehabilitation"[MeSH Terms] OR ("cardiac"[All Fields] AND "rehabilitation"[All Fields]) OR "cardiac rehabilitation"[All Fields]) | 28416 |
| --- | --- | --- | --- |
|  | Acute decompensated heart failure | #2 ("acute"[All Fields] OR "acutely"[All Fields] OR "acutes"[All Fields]) AND ("heart failure"[MeSH Terms] OR ("heart"[All Fields] AND "failure"[All Fields]) OR "heart failure"[All Fields]) | 678220 |
|  |  | #3 #1AND #2 Filters: Randomized Controlled Trial | 267 |
| EMBASE | Cardiac rehabilitation | #1 ('physical rehabilitation'/exp OR 'physical rehabilitation' OR 'exercise training' OR 'cardiac rehabilitation') AND [randomized controlled trial]/lim | 6507 |
|  | Acute decompensated heart failure | #2 ('acute heart failure'/exp OR 'acute heart failure' OR 'acute decompensated heart failure') AND [randomized controlled trial]/lim | 1234 |
|  |  | #3 #1 AND #2 | 42 |
|  |  |  |  |
| CENTRAL | Acute decompensated heart failure | #1 (acute heart failure):ti,ab,kw OR (acute decompensated heart failure):ti,ab,kw (Word variations have been searched)" in Trials (Word variations have been searched) | 9692 |
|  | Cardiac rehabilitation | (cardiac rehabilitation):ti,ab,kw OR ("physical activity"):ti,ab,kw OR (exercise training):ti,ab,kw (Word variations have been searched)" in Trials (Word variations have been searched) | 56663 |
|  |  | #3 #1 AND #2 | 334 |
| WANFANG | Cardiac rehabilitation | #1 Cardiac rehabilitation, ti, ab OR exercise trainig,ti,ab OR physical rehabilitation,ti,ab | 7595 |
|  | Acute decompensated heart failure | #2 Heart failure,ti,ab OR acute decompensated heart failure"ti,ab OR acute heart failure,ti,ab | 18629 |
|  |  | #3 #1 AND #2 | 242 |
